# Argo: enabling the development of bespoke workflows and services for disease annotation

**DOI:** 10.1093/database/baw066

**Published:** 2016-05-17

**Authors:** Riza Batista-Navarro, Jacob Carter, Sophia Ananiadou

**Affiliations:** National Centre for Text Mining, School of Computer Science, University of Manchester, Manchester, UK

## Abstract

Argo (http://argo.nactem.ac.uk) is a generic text mining workbench that can cater to a variety of use cases, including the semi-automatic annotation of literature. It enables its technical users to build their own customised text mining solutions by providing a wide array of interoperable and configurable elementary components that can be seamlessly integrated into processing workflows. With Argo's graphical annotation interface, domain experts can then make use of the workflows' automatically generated output to curate information of interest.

With the continuously rising need to understand the aetiology of diseases as well as the demand for their informed diagnosis and personalised treatment, the curation of disease-relevant information from medical and clinical documents has become an indispensable scientific activity. In the Fifth BioCreative Challenge Evaluation Workshop (BioCreative V), there was substantial interest in the mining of literature for disease-relevant information. Apart from a panel discussion focussed on disease annotations, the chemical-disease relations (CDR) track was also organised to foster the sharing and advancement of disease annotation tools and resources.

This article presents the application of Argo’s capabilities to the literature-based annotation of diseases. As part of our participation in BioCreative V’s User Interactive Track (IAT), we demonstrated and evaluated Argo’s suitability to the semi-automatic curation of chronic obstructive pulmonary disease (COPD) phenotypes. Furthermore, the workbench facilitated the development of some of the CDR track’s top-performing web services for normalising disease mentions against the Medical Subject Headings (MeSH) database. In this work, we highlight Argo’s support for developing various types of bespoke workflows ranging from ones which enabled us to easily incorporate information from various databases, to those which train and apply machine learning-based concept recognition models, through to user-interactive ones which allow human curators to manually provide their corrections to automatically generated annotations. Our participation in the BioCreative V challenges shows Argo’s potential as an enabling technology for curating disease and phenotypic information from literature.

**Database URL**: http://argo.nactem.ac.uk

## Introduction

One of the diseases of utmost concern recently is chronic obstructive pulmonary disease (COPD), a category of medical conditions characterised by blockage of the lung airways and breathing difficulties. In 2011, it was the third leading cause of death in the United States, and has been predicted to become the third one worldwide by 2030 (http://www.who.int/respiratory/copd/en).

Phenotypes are an organism’s observable traits which help in uncovering the underlying mechanisms of a patient’s medical condition. In the case of COPD, disease and clinical manifestations are heterogeneous and widely vary from one patient to another. Methods for identifying phenotypes (i.e. COPD phenotyping) have thus been adopted to allow for the well-defined characterisation of COPD patients according to their prognostic and therapeutic characteristics (1).

The task of identifying phenotypes within narratives and documents, i.e. phenotype curation, is a widely adopted practice especially within the clinical community. As the amount of relevant textual data (e.g. clinical records and scientific literature) has continued to grow at an increasingly fast pace, substantial time and effort are required from human experts in curating phenotypic information. Aiming to alleviate this burden on human experts, we developed text mining workflows for semi-automatic phenotype curation in our Web-based workbench, Argo (2). To demonstrate and evaluate Argo's suitability for the task, we participated in the User Interactive Task (IAT) of BioCreative V, enlisting the help of five experts who volunteered to curate COPD phenotypes from full-text documents. Three subtasks were carried out: (1) the markup of phenotypic mentions in text, i.e. concept recognition, (2) linking of mentions to relevant databases, i.e. normalisation and (3) annotation of relations between COPD and other concepts. Results from the effort indicate that Argo shows promise as a phenotype curation tool.

Furthermore, our methods for concept recognition and normalisation were officially evaluated against the benchmark data sets of the Disease Named Entity Recognition and Normalisation (DNER) subtask of BioCreative V’s Chemical-Disease Relations (CDR) track, which called for the development of web services capable of automatically recognising disease mentions in scientific abstracts, and assigning to them identifiers from the Medical Subject Headings (MeSH) database (3).

We note that this article consolidates and extends our reported work in the BioCreative V workshop. In the remainder of this paper, we shall first provide the reader with an overview of Argo’s capabilities which support biocuration and then proceed to describing in detail the annotation tasks at hand. The methods that we employed in developing our text mining workflows and services are described next, followed by a discussion of how they were evaluated. We conclude by summarising our contributions and by providing some insights on our plans for future work.

## System features

Argo is a generic text mining (TM) framework. Rather than catering to a specific application or use case, it enables its technical users to build their own customised TM solutions by providing a wide array of interoperable and configurable elementary components that can be seamlessly integrated into processing pipelines, i.e., workflows. We outline below the various features of Argo which enable its biocuration capabilities.

### Web-based availability

Developed as a Web application, Argo does not require its users to perform any software installation, and can be accessed using any of the following browsers: Google Chrome, Mozilla Firefox and Safari. All workflows are executed on a remote server and can proceed even when users close the application. Argo also supports high-throughput text processing by providing an option to execute workflows on computing clusters, e.g. Amazon Elastic Compute Cloud (EC2), HTCondor. The interface displays a listing of a user's currently running workflows to allow for progress monitoring.

### Library of interoperable components

Key to Argo's processing capabilities is its continuously growing library of elementary processing tools. Owing to their compliance with the industry-accepted Unstructured Information Management Architecture (UIMA) (4), these interoperable components can interface with each other and when combined into meaningful workflows, can form tailored TM solutions that address specific tasks. Each component in the library plays any one of three roles. *Readers* are for loading input data, e.g. document collections, either from a user's own files or from external resources (e.g. PubMed). With readers for a variety of data formats such as plain text, tab-separated values (TSV), XML (e.g. BioC and XMI) and RDF, Argo enables its workflows to deserialise data from many publicly available corpora. Meanwhile, *Analytics* are implementations of various natural language processing (NLP) methods, and enrich input text with annotations at the lexical (e.g. lemmatisers), syntactic (e.g. tokenisers, dependency parsers) and semantic (e.g. named entity recognisers, concept normalisers and event extractors) levels. Finally, *Consumers* facilitate the serialisation of annotations to any of a user's preferred output formats. As with readers, Argo provides a wide selection of data encodings to choose from, including BioC, XMI, RDF and TSV. The library of components is accessible to the user through Argo’s interface for designing workflows, which we describe next.

### Workflow designer

**Figure 1.**
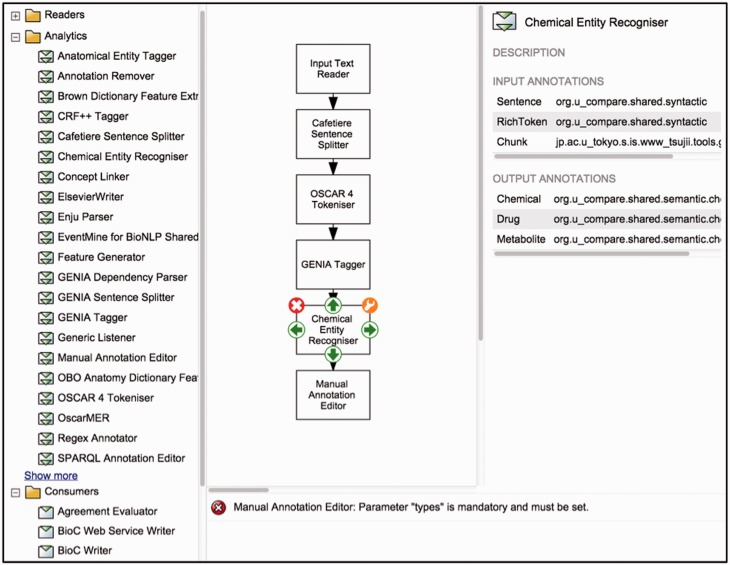
Argo’s interface for designing workflows. The left panel displays the library of components whilst the block diagramming canvas at the centre allows for arranging and interconnecting selected components.

To support the creation of customised workflows out of the components previously described, Argo provides a block diagramming interface for graphically constructing TM workflows (Figure 1). The library (described above) is displayed as a list of components (sorted alphabetically by name), which can be grouped according to their role or the annotation schema they follow. A user designs a workflow by selecting components from the library, which will appear on the canvas as blocks. To define the processing sequence, the user arranges these blocks in the desired order and interconnects them using the available connection ports. Each of the components can then be customised with user-supplied parameter values. Guiding the user are detailed descriptions of each component's input and output types, as well as a panel that displays warning messages if problematic issues with a workflow have been detected. A tutorial (http://argo.nactem.ac.uk/tutorials/creating-workflows/) demonstrating the steps for creating and running workflows is also provided in Argo’s webpage.

### Manual and automatic modes of annotation

**Figure 2.**
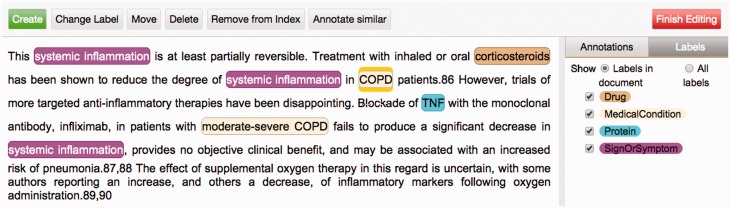
Argo’s Manual Annotation Editor. The graphical interface serves as a visualisation tool, with colourcoded filters for narrowing down the types of annotations displayed. More importantly, it provides buttons that allow users to create, remove and manipulate annotations which can be either simple (e.g. text spanbased) or structured (e.g. relations or events).

**Figure 3.**
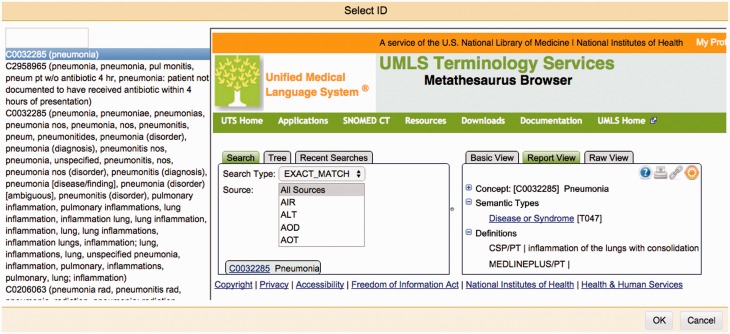
The Manual Annotation Editor’s interactive normalisation interface. On the left-hand side is a ranked list of best matching candidates for a given mention. The right-hand side panel allows users to search the external database themselves, to assist them in selecting an identifier that will be assigned to a mention.

One of Argo's available components is the Manual Annotation Editor which provides access to a graphical interface for manipulating annotations (Figure 2). To add new text span-based annotations, users highlight relevant tokens and assign suitable labels; annotated text spans are displayed according to an in-built colour-coding scheme. Structured annotations (e.g. relations, events) can be added by creating template-like structures and filling the slots either with primitive values or with any of existing text span annotations. Annotators can also remove annotations or modify the span, label or any other attribute value of existing annotations. Assignment of unique identifiers from external databases (e.g. for normalisation) is especially supported in Argo through an interactive utility for disambiguation that automatically retrieves a ranked list of matching candidates and displays further information coming directly from the relevant resource (Figure 3).

Argo supports different modes of annotation. For purely manual annotation, a workflow that consists only of a reader, the Manual Annotation Editor and any of the available consumers for saving annotations will suffice. In cases where text mining support is desired, we need to define to what extent we require the automation by incorporating chosen TM components into the workflow, before the Manual Annotation Editor. A curator can then use the Editor to revise the automatically generated annotations or supply his/her own new ones. It is also possible to visualise and revise annotated documents directly from a user's document space. This feature was incorporated into Argo to make it more convenient for annotators to review their previously annotated documents.

## Curation tasks

The annotation effort was comprised of three information extraction subtasks pertinent to the curation of COPD phenotypes.

### Markup of phenotypic mentions

The first subtask called for the demarcation of expressions denoting COPD phenotypes, which were also assigned semantic labels by the curators. Following the recent recommendation by Barker and Brightling (5) who argued that a multi-scale approach integrating information from various dimensions (e.g. gene, cell, tissue, organ) is necessary in order to fully understand a COPD patient's condition, we captured phenotypes falling under any of the categories: (i) medical condition: a disease or medical condition, including COPD comorbidities, (ii) sign or symptom: an observable irregularity manifested by a patient, (iii) protein and (iv) drug.

### Normalisation of mentions

Many phenotypic concepts can be expressed in text in numerous ways. The phenotype pertaining to blockage of lung airways, for example, can take the form of any of the following variants and more: *airways are blocked*, *blocked airways*, *blockage of airways*, *airways obstruction*, *obstructed lung airways*. As a means for homogenising variants, the normalisation of surface forms to corresponding entries in ontologies was also required by our curation task. The following resources were leveraged: the Unified Medical Language System (UMLS) (6) for normalising mentions of medical conditions and signs or symptoms, UniProt (7) for proteins and Chemical Entities of Biological Interest (ChEBI) (8) for drugs.

### Relation annotation

The last subtask involved the annotation of binary relations between a COPD mention and any other concept falling under our semantic categories of interest. This subtask is aimed at capturing information addressing the following questions:

- Which other medical conditions (e.g. comorbidities) are associated with COPD?
- Which signs or symptoms are indicative of COPD?
- Which genes or proteins underlie the mechanisms of COPD?
- Which drugs affect COPD?

To assist our curators in accomplishing the tasks, specifications and training material were provided. Firstly, annotation guidelines and detailed instructions were published as web pages, linked from Argo's main page (http://argo.nactem.ac.uk/tutorials/curation-of-copd-phenotypes). A screencast demonstrating the use of Argo’s annotation interface was also prepared (http://youtu.be/uOjwgmaXk00). Furthermore, one-to-one tutorials were offered.

Based on the recommendation of the IAT organisers, the curators were requested to spend a total of at least four hours on the effort, distributed over two weeks; this excludes the time spent on familiarising themselves with the system and annotation guidelines. During the first week, they were asked to accomplish the first and second subtasks, i.e. marking up of phenotypic mentions and linking them to ontologies (Phase 1). The second week was then dedicated to the annotation of relations between concepts annotated during the preceding week (Phase 2). For each week, the curators provided their annotations in two modes. In the first mode, they were required to create annotations completely manually, i.e. without any TM support. In the second mode, meanwhile, they were given TM support in the form of automatically generated annotations. In the next section, we describe in detail the workflows that were developed to facilitate the text mining-assisted mode of the curation tasks.

## Text mining workflows

As described above, the phenotype curation task was logistically carried out in two phases: Phase 1 which was focussed on the marking up of mentions and their normalisation, and Phase 2 in which the associations between the mentions resulting from the previous phase were captured. A semi-automatic curation workflow was prepared for each phase.

### Concept recognition and normalisation workflow

**Figure 4.**
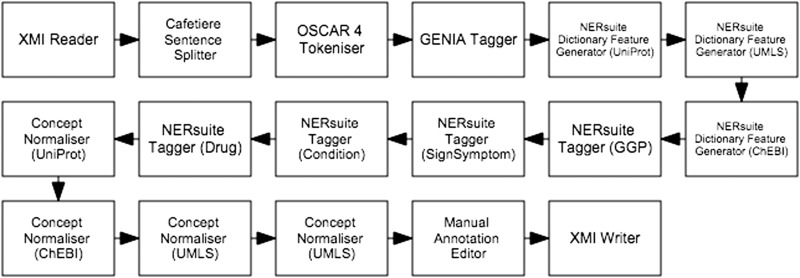
Semi-automatic workflow for text mining-assisted COPD concept curation. It accomplishes both of the concept recognition and normalisation subtasks.

The first workflow, depicted in Figure 4, performs machine learning-based recognition of concepts followed by normalisation according to string similarity heuristics. We cast concept recognition as a sequence labelling task, in which individual tokens of any given text are assigned labels according to the begin-inside-outside (BIO) encoding scheme. To this end, our documents (which are loaded by the XMI Reader component) were first segmented into sentences by the Cafetiere Sentence Splitter (http://tinyurl.com/hv9m9tg). These in turn were decomposed into tokens by the OSCAR4 Tokeniser (9) which were then assigned lemmatised forms as well as part-of-speech (POS) and chunk tags by the GENIA Tagger (10).

We employed the NERsuite package (http://nersuite.nlplab.org), an implementation of conditional random fields (CRFs) (11), to apply pre-trained models for sequence labelling. Each token was presented to the CRFs as a rich set of lexical, orthographic and semantic features, such as:

1. two, three and four-character *n*-grams
2. token, POS tag and lemma unigrams and bigrams within a context window of three
3. presence of digits or special characters
4. a flag indicating that a token contains only uppercase letters
5. word shape, with all of a token’s uppercase letters converted to ‘A’, lowercase letters converted to ‘a’, digits to ‘0’ and special characters to ‘_’
6. matches against semantically relevant dictionaries.

**Figure 5.**
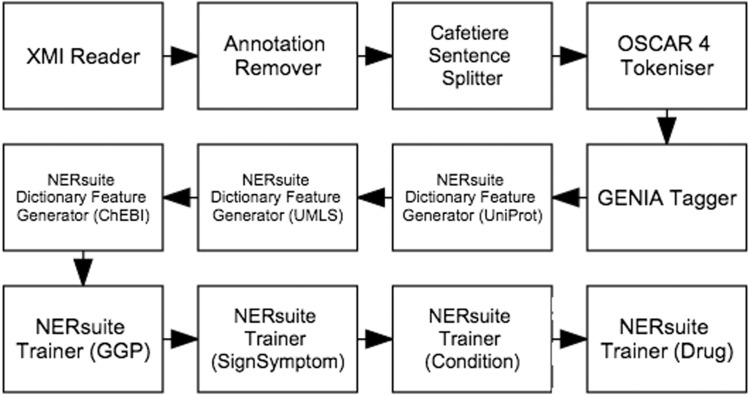
Workflow for training conditional random fields (CRF) models to recognise COPD concepts. For each of our concept types of interest, a model was produced by the corresponding NERsuite Trainer component.

Whilst the first five types of features were based on results of the pre-processing components described above, the dictionary matches were extracted by the NERsuite Dictionary Feature Generator. It can be seen from Figure 4 that an instance of this component is required for each dictionary of interest, i.e. one for each of UniProt (for proteins), UMLS (for medical conditions and signs/symptoms) and ChEBI (for drugs). These components are then succeeded by a series of NERsuite Tagger components—one for each of our mention types of interest—which assign BIO labels to the input tokens based on the predictions of user-specified CRF models, pre-trained by a separate Argo workflow, depicted in Figure 5. We note that whilst this is quite similar to the concept recognition workflow (Figure 4), it instead makes use of NERsuite Trainer components in order to produce CRF models which have learnt the token features presented to it.

The result of the tagger components are mentions with semantic type labels. These are then subsequently assigned identifiers from respective databases by our Concept Normaliser components, which were developed using unsupervised approaches based on string similarity measures. Each of the Concept Normaliser components was supplied with a precompiled dictionary. The first step in the preparation of such a dictionary is the collection of names (including synonyms) and corresponding identifiers from the database of interest. Each of the names was then converted to a canonical form based on the following series of steps:

1. Converting all characters to lowercase
2. Removal of stop words and punctuation
3. Stemming of each remaining token
4. Alphabetical re-ordering of tokens

This process results in the generation of a new dictionary containing the equivalent canonical forms of names (and their corresponding identifiers) from the relevant database. Each mention requiring normalisation undergoes the same process of canonicalisation. The resulting canonical form is then used to query the relevant compiled dictionary for the most similar strings according to the Jaro-Winker distance measure (12). All entries in the dictionary whose similarity score is above a predefined threshold of 0.80 are considered candidates. Since multiple candidates having the same score were being returned, we additionally applied the Levenshtein distance measure in order to compute the similarity between the query name and a candidate. This allowed us to induce a more informative ranking of the candidates, from which the topmost result was considered as the best matching dictionary entry. The identifier attached to this entry is finally assigned to the name in question. It is worth noting at this point that further strategies for normalising disease mentions were explored as part of our contribution to the BioCreative V’s CDR track, described in a later section.

After executing the normalisation components, Argo will prompt the user to launch the Manual Annotation Editor and supply their corrections. Once the user’s completion of his/her annotations, the annotated documents are finally saved as XMI files by the XMI Writer component.

### Relation annotation workflow

**Figure 6.**
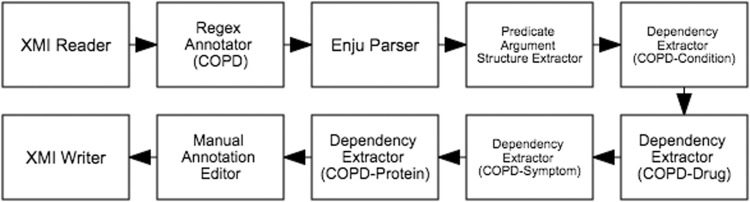
Semi-automatic workflow for text mining-assisted relation annotation. Relations between COPD and other concept types are automatically extracted based on syntactic dependencies.

The semi-automatic workflow for the text mining-assisted mode of Phase 2 is depicted in Figure 6. It begins with an XMI Reader which loads the annotated documents from Phase 1. Since the relationships that we aim to capture in this subtask are centred around COPD, the Regex Annotator component was incorporated into the workflow to identify mentions denoting COPD (e.g. ‘chronic obstructive pulmonary disease’, ‘COPD’, ‘copd’). Deep syntactic parsing is performed by the next component, Enju Parser (13), whose results are encoded as predicate-argument structures (PASes) by the Predicate Argument Structure Extractor. Each COPD mention is then paired with another mention along a dependency path by the Dependency Extractor. These mention pairs form the binary relations which are presented by the Manual Annotation Editor to the expert for corrections. The validated annotations are finally saved in XMI files by the XMI Writer.

## Optimising disease name normalisation

Further normalisation strategies were explored as part of our participation in the CDR track of BioCreative V, which called for the development of disease recognition and normalisation methods deployed as web services complying with either the PubTator (14) or BioC (15) format. Although in the end our web services were hosted outside of the Argo environment, the workbench still served as our development platform by facilitating the training of disease name recognition models on a new corpus conveniently.

As our baseline method, a dictionary of disease names/synonyms (and corresponding identifiers) from MeSH was compiled, according to the method described in the previous section. Building upon this method, we proposed another approach which is based on the incorporation of further semantics. Firstly, two corpora, namely the official CDR corpus (16) provided by the track organisers and the NCBI Disease Corpus (17), were used as sources of variants actually used in scientific literature which were added to our MeSH dictionary by cross-referencing provided gold standard identifiers. For example, the exact mention ‘brain damage’ is assigned the MeSH identifier ‘D001930’ in the CDR development corpus but does not appear as a name or synonym in MeSH. Hence, to increase the coverage of our own MeSH dictionary, we expand it by adding an entry for ‘D001930:brain damage’. Table 1 presents the resulting size of our different MeSH dictionary versions after the application of this method.

**Table 1.** Number of unique names in each version of the MeSH dictionary we compiled. Only entries under the Diseases and Psychiatry/Psychology subtrees of MeSH were included

Source	Number of entries
MeSH	53,839
MeSH + CDR(training + development)	55,315
MeSH + CDR(training + development) + NCBI(all)	56,596

Secondly, we compiled a list of medical root words (http://www.stedmansonline.com/webFiles/Dict-Stedmans28/APP05.pdf) and automatically combined them with affixes that are synonymous with terms pertaining to medical disorder such as ‘disease’, ‘deficiency’, ‘inflammation’, to generate potential variants that can be then matched against MeSH. If the score of the best matching candidate retrieved for a mention using string similarity is lower than a predefined threshold, the mention is checked for the occurrence of medical root words. The word ‘neuropathy’, e.g. is broken down into ‘neuro’ (nerve or nervous) and ‘pathy’ (disease), based on which our method automatically generates ‘nervous disease’. When used to query our own compiled MeSH dictionary, ‘nervous disease’ fetches ‘nervous system disease’, thus leading to the assignment of the correct identifier to “neuropathy”.

## Results and discussion

In this section, we first present our results from the COPD phenotype curation effort, and then shift the focus of the discussion to our performance in the disease name normalisation task.

A total of five human experts volunteered to participate in our COPD phenotype curation task. As mentioned previously, upon the recommendation of the IAT organisers, each of them was asked to spend at least 4 h on the full task. Our suggestion was to allocate the first two hours on Phase 1 (mention recognition and normalisation) and the remaining two on Phase 2 (relation annotation). A goal of IAT is to evaluate the benefits gained from automating part of the curation process using text mining (TM) techniques; to this end, for each of our two phases, each annotator was asked to spend at least an hour on non-TM-assisted and another hour on TM-assisted curation.

A corpus of 30 COPD-relevant PubMed Central Open Access papers that we have previously developed (18) was exploited in this effort. The corpus was split into two subsets with 15 papers each: one for training the machine learning-based models underpinning the automatic COPD concept annotation workflow, and the other from which the documents for curation were drawn. Since the time constraints did not make the annotation of entire full-text papers feasible, we defined a document as a smaller chunk of text (e.g. section paragraphs according to each paper's metadata). Based on automatic random selection, 124 such documents were set aside for the curation task. The first 62 were used for purely manual curation while the remaining were exploited in the text mining-assisted mode of the task. All of the curators were asked to annotate the same data set.

For concept annotation (i.e. marking up phenotypic mentions and linking them to ontologies), the experts completed the curation of an average of nine passages in an hour. In the TM-assisted mode of concept annotation, the rate increased to an average of 14 passages per hour. Relation annotation was less time-consuming: in an hour, the curators annotated relations in 25 and 35 passages, in the non-TM and TM-assisted modes of annotation, respectively. The curators were asked to annotate the passages in the same order that Argo displayed them, i.e. alphabetically by file name. In this way, even if the curators were carrying out their annotations at different rates (some curating more passages than the others within the allocated time), we were able to compile a corpus of 20 passages which were commonly annotated by all five curators.

We estimated inter-annotator agreement (IAA) based on concept annotations (i.e. text span boundaries and semantic categories) manually produced for this set. We measured the F-score between each of the 10 pairs of annotators and obtained an average of 68.12%. The lowest agreement is 49.84% while the highest is 82.78%.

Using the concept annotations (i.e., text span boundaries and semantic types) of an expert who voluntarily curated all of the 124 passages in the data set, we evaluated the performance of the concept annotation workflow which formed the basis of the text mining support provided to the curators. The per-category and overall results of this evaluation are presented in Table 2. It can be observed that the highest performance was obtained for the medical condition/disease category but the results are quite poor for signs/symptoms. This poor performance can be attributed to the sparsity of sign/symptom samples in our training corpus. Out of a total of 5611 mentions in the training set, only 934 correspond to signs/symptoms (whilst there are 2544 mentions of medical conditions). It is worth noting, however, that the overall precision, recall and F-score values obtained are 68.17, 63.96 and 66.97%, respectively. These results are quite encouraging especially considering that the F-score (66.97%) is very close to the measured IAA (68.12%), indicating that our automatic concept annotation workflow performs comparably with human curators.

**Table 2.** Evaluation of the automatic concept annotation workflow. Results are presented in terms of micro-averages over the 124 full paper sections in our corpus.

	Precision	Recall	*F*-score
Sign or symptom	42.67	26.67	32.82
Protein	73.33	55.00	62.86
Drug	65.24	71.01	68.00
Medical condition	75.52	69.88	72.59
Overall	68.17	63.92	65.97

Further evaluation of our disease name recognition and normalisation methods were carried out, based on the CDR benchmark data sets provided by the track organisers. For recognition, using a CRF model trained on the CDR training and development sets (consisting of a total of 1000 PubMed abstracts), we obtained an F-score of 84.39% (precision  =  87.67% and recall  =  81.35%) on the CDR development set (with 500 abstracts), according to the evaluation library provided.

Three different versions of our disease name normalisation approach have been officially evaluated on the CDR Test corpus of 500 abstracts, the results of which are presented in Table 3. All of them exploited the automatic Greek/Latin medical root/affix translation technique, although using different thresholds in determining whether the translation should be carried out. In the first version (Run 1), the translation is performed only if the string similarity between the mention in question and the topmost candidate is below a threshold of 0.92 (optimised for recall). It made use of a version of MeSH that included only mentions from the CDR Training and Development sets. Both the second (Run 2) and third (Run 3) versions leveraged a MeSH dictionary that additionally incorporated mentions from the NCBI Disease Corpus. A threshold of 0.94 was applied in Run 2 while 0.96 was used in Run 3 (optimised for precision). We wrapped our methods as a web service that accepts and outputs data in the BioC format. It is available for public use (http://nactem.ac.uk/biocreative/dner?format=bioc&run=x where x can be any of 1, 2 or 3) and allows for any of the three versions to be invoked. According to the official evaluation carried out by the CDR task organisers over the 40 submissions from 16 different teams, our services obtained the third best F-score (85.56%) and the best precision amongst the three top-performing teams.

**Table 3.** Evaluation of our disease name normalisation approaches on the CDR test corpus.

Run	Dictionary	Threshold for translating medical affixes	Precision	Recall	*F*-score
Run 1	MeSH + CDR(training + development)	0.92	88.89	82.14	85.39
Run 2	MeSH + CDR (training + development)+ NCBI(all)	0.94	89.51	81.94	85.56
Run 3	MeSH + CDR(training + development)+ NCBI(all)	0.96	89.89	81.44	85.46

## Related work

In this section, we compare our work with other existing tools and approaches, including those reported in BioCreative V. Firstly, we provide a discussion of the similarities and differences between Argo and other curation systems in terms of functionality and performance. This is then followed by a comparison of our proposed disease name normalisation methods with other approaches.

In terms of functionality, Argo is similar to some of the other systems which were also showcased in the IAT track of BioCreative V. Like Argo, the following systems demonstrated their capabilities for automatic concept annotation and relation extraction: Egas (19), GenDisFinder (20), MetastasisWay (21) and BELIEF (22). It is worth noting that Egas is the most similar system to Argo. Whilst the other three were developed to cater to specific tasks (gene-disease association extraction for GenDisFinder, metastasis pathway construction for MetastasisWay, and generation of Biological Expression Language statements for BELIEF), Egas—like Argo—is a generic curation platform that allows users to define new tasks through the custom configuration of underlying tools and annotation schema. We also note that out of these four other systems, only Egas reported on their calculated inter-annotator agreement for the concept annotation task, i.e. 74%. However, the performance of their automatic tools against gold standard data was not reported, whereas that of Argo’s concept annotation workflow (F-score  =  66.97%) was shown to approximate the measured agreement between human curators for the same task (68.12%).

Disease name normalisation was not a well explored task until the specifications of the CDR task were defined as part of BioCreative V. Whilst some generic normalisation tools such as NCBO Annotator (23), MetaMap (24) and ConceptMapper (25) have been applied to this task, only DNorm (26) was proposed to specifically address the problem of linking disease names to relevant lexica. The CDR task, however, fostered the development of new state-of-the-art disease normalisation tools, e.g. the system from the National Cheng Kung University (NCKU) (27) and LeadMine (28), with which we have compared our own work on the CDR task, in terms of proposed methods as well as performance.

Both of the NCKU and LeadMine systems are similar to our normalisation approach, in that we all compiled comprehensive disease name dictionaries by leveraging various sources. Whereas we augmented MeSH with the disease names annotated in the CDR and NCBI Disease gold standard corpora, NCKU’s system additionally incorporated names from the MEDIC dictionary (29). Meanwhile, LeadMine combined MeSH entries with names from the Disease Ontology (30) and Wikipedia. These systems, like ours, made use of string similarity techniques to match disease mentions in text with entries in the respective compiled dictionaries. However, our strategies for pre-processing disease mentions and dictionary entries (e.g. stop-word removal, stemming) are more sophisticated, with some similarities with the ConceptMapper’s approach. Furthermore, none of the other systems explored the resolution of medical root words as we did.

Quite unique amongst all of the disease name normalisation systems mentioned is DNorm, which was implemented based on a pairwise learning-to-rank algorithm. In terms of performance on the CDR Test data, however, DNorm was not very competitive with an F-score of 80.64%, whereas NCKU’s system, LeadMine and our own approach obtained higher F-scores of 86.46%, 86.12% and 85.56%, respectively – the best results according to the official CDR task evaluation.

## Conclusion

We have described in this article our recent contributions to disease annotation. Firstly, as an outcome of our participation in BioCreative V, the component library of our Argo workbench has been further enriched with components which enable the straightforward training of machine learning-based models as well as interfacing with disease-specific resources, e.g. UMLS and MeSH. As a consequence, the workbench facilitated the development of text mining workflows and services customised for disease phenotype curation. Results of the evaluation of the benefit of using Argo as a curation platform show that its semi-automatic workflows, for example, facilitated the curation of COPD phenotypes in ∼50% more documents. Furthermore, the overall F-score obtained by the concept annotation workflow closely approximates the inter-annotator agreement amongst five experts, indicating that the workflow performs comparably with a human. In terms of the recognition and normalisation of disease mentions alone, we have demonstrated that we can obtain F-scores as high as 85% upon optimisation of our strategies.

One of our immediate steps after this work is the integration of the literature-curated disease information with knowledge bases, e.g. PhenomeNet. This would allow for the evaluation of how much this type of information could contribute towards the discovery of new disease-relevant knowledge.

## References

[1] Han M.K. Alvar A. Calverley P.M. (2010) Chronic obstructive pulmonary disease phenotypes. Am. J. Respir. Crit. Care Med., 182, 598–604. 20522794 10.1164/rccm.200912-1843CC PMC6850732

[2] Rak R. Rowley A. Black W. (2012) Argo: an integrative, interactive, text mining-based workbench supporting curation. Database, 2012. 10.1093/database/bas010 PMC3308166 22434844

[3] Lipscomb C.E. (2000) Medical Subject Headings (MeSH). Bull. Med. Libr. Assoc., 88, 265–266. 10928714 PMC35238

[4] Ferrucci D. Lally A. (2004) UIMA: An architectural approach to unstructured information processing in the corporate research environment. Nat. Lang. Eng., 10, 327–348.

[5] Barker B. Brightling C. (2013) Phenotyping the heterogeneity of chronic obstructive pulmonary disease. Clin. Sci., 124, 371–387. 23190267 10.1042/CS20120340

[6] Bodenreider O. (2004) The Unified Medical Language System (UMLS): integrating biomedical terminology. Nucleic Acids Res., 32, D267–D270. 14681409 10.1093/nar/gkh061 PMC308795

[7] UniProt. (2014) Activities at the Universal Protein Resource (UniProt). Nucleic Acids Res., 42, D191–D198. 24253303 10.1093/nar/gkt1140 PMC3965022

[8] Hastings J. de Matos P. Dekker A. (2012) The ChEBI reference database and ontology for biologically relevant chemistry: enhancements for 2013. Nucleic Acids Res, 41, D456–D463. 23180789 10.1093/nar/gks1146 PMC3531142

[9] Jessop D.M. Adams S.E. Willighagen E.L. (2011) OSCAR4: a flexible architecture for chemical text-mining. J. Cheminform., 3, 41. 21999457 10.1186/1758-2946-3-41 PMC3205045

[10] Tsuruoka Y. Tateisi J.D. Kim Y. Developing a robust part-of-speech tagger for biomedical text In: Advances in Informatics - 10th Panhellenic Conference on Informatics, vol. 3746 Springer, Volos, Greece, pp. 382–392.

[11] Lafferty A. McCallum F.C.N. Pereira J.D. Conditional Random Fields: Probabilistic Models for Segmenting and Labeling Sequence Data. In: Proceedings of the Eighteenth International Conference on Machine Learning, 2001, pp. 282–289.

[12] Jaro M.A. (1989) Advances in record-linkage methodology as applied to matching the 1985 Census of Tampa, Florida. J. Am. Stat. Assoc, 84, 414–420.

[13] Miyao Y. Tsujii J. (2008) Feature forest models for probabilistic hpsg parsing. Comput. Linguist., 34, 35–80.

[14] Wei C.H. Kao H.Y. Lu Z. (2013) PubTator: a web-based text mining tool for assisting biocuration. Nucleic Acids Res., 41, W518–W522. 23703206 10.1093/nar/gkt441 PMC3692066

[15] Comeau D.C. Islamaj Doğan R. Ciccarese P (2013) BioC: a minimalist approach to interoperability for biomedical text processing. Database, 2013. 10.1093/database/bat064 PMC3889917 24048470

[16] Li Y. Sun R.J. Johnson J. Annotating chemicals, diseases, and their interactions in biomedical literature. In: Proceedings of the Fifth BioCreative Challenge Evaluation Workshop, 2015, pp. 173–182.

[17] Doğan R.I. Leaman R. Lu Z. (2014) NCBI disease corpus: A resource for disease name recognition and concept normalization. J. Biomed. Inform., 47, 1–10. 24393765 10.1016/j.jbi.2013.12.006 PMC3951655

[18] Fu X. Batista-Navarro R. Rak R. (2015) Supporting the annotation of chronic obstructive pulmonary disease (COPD) phenotypes with text mining workflows. J. Biomed. Semantics, 6, 8. 25789153 10.1186/s13326-015-0004-6 PMC4364458

[19] Matos D. Campos R. Pinho S. Assisted mining and curation of genomic variants using Egas. In Proceedings of the Fifth BioCreative Challenge Evaluation Workshop, 2015, pp. 396–402.

[20] Subramani J. Natarajan S. (2015) An integrated text mining system based on network analysis for knowledge discovery of human gene-disease associations (GenDisFinder). In Proceedings of the Fifth BioCreative Challenge Evaluation Workshop, pp. 427–434.

[21] Dai C.H. Su P.T. Lai H.J. (2015) MET pathway in PubMed: a pathway visualization and curation system. In Proceedings of the Fifth BioCreative Challenge Evaluation Workshop, pp. 418–426.

[22] Madan S. Hodapp J. Fluck S. (2015) BELIEF Dashboard – a Web-based curation interface to support generation of BEL networks. In Proceedings of the Fifth BioCreative Challenge Evaluation Workshop, pp. 409–417.

[23] Jonquet C. Shah N.H Musen M.A. (2009) The Open Biomedical Annotator. Summit on Translat. Bioinforma. 56–60. 21347171 PMC3041576

[24] Aronson A.R Lang F.M. (2010) An overview of MetaMap: historical perspective and recent advances. J. Am. Med. Inform. Assoc, 17, 229–236. 20442139 10.1136/jamia.2009.002733 PMC2995713

[25] Tanenblatt M. Coden A. Sominsky I. (2010) The ConceptMapper approach to named entity recognition. In: Proceedings of Language Resources and Evaluation Conference (LREC) 2010, pp. 546–551.

[26] Leaman R. Islamaj Doğan R. Lu Z. (2013) DNorm: disease name normalization with pairwise learning to rank. Bioinformatics, 29, 2909–2917. 23969135 10.1093/bioinformatics/btt474 PMC3810844

[27] Lee Y.Y Hsu H.Y. Kao H.C. (2015) An enhanced CRF-based system for disease name entity recognition and normalization on BioCreative V DNER Task. In Proceedings of the Fifth BioCreative Challenge Evaluation Workshop, pp. 226–233.

[28] Lowe D.M. O'Boyle N.M. Sayle R.A. (2015) LeadMine: Disease identification and concept mapping using Wikipedia. In Proceedings of the Fifth BioCreative Challenge Evaluation Workshop, pp. 240–246.

[29] Davis A.P. Wiegers T.C. Rosenstein M.C (2012) MEDIC: a practical disease vocabulary used at the Comparative Toxicogenomics Database. Database. 2012. 10.1093/database/bar065 PMC3308155 22434833

[30] Schriml L.M. Arze C. Nadendla S. (2012) Disease Ontology: a backbone for disease semantic integration. Nucl. Acids Res., 40, D940–D946. 22080554 10.1093/nar/gkr972 PMC3245088

